# Biochemical and structural characterization of beta-carbonic anhydrase from the parasite *Trichomonas vaginalis*

**DOI:** 10.1007/s00109-021-02148-1

**Published:** 2021-10-15

**Authors:** Linda J. Urbański, Andrea Angeli, Vasyl V. Mykuliak, Latifeh Azizi, Marianne Kuuslahti, Vesa P. Hytönen, Claudiu T. Supuran, Seppo Parkkila

**Affiliations:** 1 Faculty of Medicine and Health Technology, Tampere University, Arvo Ylpön katu 34, 33520 Tampere, Finland; 2 Neurofarba Department, Sezione Di Chimica Farmaceutica E Nutraceutica, Università Degli Studi Di Firenze, Via U. Schiff 6, 50019 Sesto Fiorentino (Firenze), Italy; 3 Fimlab Ltd, Tampere University Hospital, Arvo Ylpön katu 4, 33520 Tampere, Finland

**Keywords:** Beta-carbonic anhydrase, Trichomonas vaginalis, Protozoan, Kinetics, Inhibition, Homology modeling

## Abstract

**Abstract:**

*Trichomonas vaginalis* is a unicellular parasite and responsible for one of the most common sexually transmittable infections worldwide, trichomoniasis. Carbonic anhydrases (CAs) are enzymes found in all lifeforms and are known to play a vital role in many biochemical processes in organisms including the maintenance of acid–base homeostasis. To date, eight evolutionarily divergent but functionally convergent forms of CAs (α, β, γ, δ, ζ, η, θ, and ι) have been discovered. The human genome contains only α-CAs, whereas many clinically significant pathogens express only β-CAs and/or γ-CAs. The characterization of pathogenic β- and γ-CAs provides important knowledge for targeting these biomolecules to develop novel anti-invectives against trichomoniasis. Here, we report the recombinant production and characterization of the second β-CA of *T. vaginalis* (TvaCA2). Light scattering analysis revealed that TvaCA2 is a dimeric protein, which was further supported with in silico modeling, suggesting similar structures between TvaCA2 and the first β-CA of *T. vaginalis* (TvaCA1). TvaCA2 exhibited moderate catalytic activity with the following kinetic parameters: *k*_cat_ of 3.8 × 10^5^ s^−1^ and *k*_cat_/*K*_*M*_ of 4.4 × 10^7^ M^−1^ s^−1^. Enzyme activity inhibition was studied with a set of clinically used sulfonamides and sulfonamide derivates. Twenty-seven out of the 39 compounds resulted in inhibition with a nanomolar range. These initial results encourage for future work entailing the design of more potent inhibitors against TvaCA2, which may provide new assets to fight trichomoniasis.

**Key messages:**

• Protozoan parasite *Trichomonas vaginalis* has two β-carbonic anhydrases (TvaCA1/2).

• TvaCA1/TvaCA2 represents promising targets for antitrichomonal drug development.

• TvaCA2 is a dimer of 20.3 kDa and possesses moderate catalytic activity.

• The most efficient inhibitor was clinical drug acetazolamide with *K*_I_ of 222.9 nM.

• The 39 tested sulfonamides form the basis for the design of more potent inhibitors.

**Supplementary Information:**

The online version contains supplementary material available at 10.1007/s00109-021-02148-1.

## Introduction

Today, we live in an era with antibiotic-resistant microbes, which will continue to cause devastating problems unless new drugs are found. One of the promising novel biomolecular drug targets is a group of metalloenzymes called carbonic anhydrases (CAs) which catalyze the interconversion between water and carbon dioxide to protons and bicarbonate ions. This chemical reaction is a fundamental part of acid–base balance in all living creatures. CAs have also been identified as a probable key factor of the molecular machinery involved in the metabolic pathways of clinically significant pathogens [1]. CAs are present in living organisms as eight isoforms: α, β, γ, δ, ζ, η, θ, and ι [2]. Humans have only the α-forms in their genome, whereas many pathogens express only β- and/or γ-CAs. This is an important discovery and makes the CAs of pathogens attractive novel and specific biomolecular drug targets. By inhibiting the enzymatic function of pathogen-specific CAs, it is possible to affect the viability and pathogenicity of the target organism. Such results have been established with, for example, *Leishmania donovani chagasi*, *Mycobacterium marinum*, and vancomycin-resistant *Enterococci* [1, 3, 4]. Genetically divergent CAs have unique 3D structures, kinetics, and inhibition and activation profiles. The unique properties of the CA enzyme families can facilitate the development of novel inhibitor compounds with a more specific mode of action. However, the first steps of inhibition studies often involve series of simple aromatic/heterocyclic sulfonamide derivates including several clinically approved drugs that have long been used to treat diseases, such as glaucoma [5] and epilepsy [6].

*Trichomonas vaginalis* is a single-cell opportunistic parasite infecting the urogenital area of men and women [7]. This disease is considered one of the most significant sexually transmittable infections (STIs) worldwide. In 2016, the World Health Organization estimated 156 million new trichomoniasis cases emerging annually, accounting for nearly half of the total STI acquisitions [8]. Even though its symptoms may not be severe [7], the harm it causes require multiple rounds of antibiotics that can make the parasite more tolerant to the drug. Antibiotic-resistant *T. vaginalis* will pose a real threat unless novel medications are discovered. Additionally, mild symptoms result in a more effective transmittance of the infection, and individuals are further exposed to more severe infections and diseases, such as prostate cancer and human immunodeficiency virus (HIV) [9, 10]. Trichomoniasis has also been associated with adverse pregnancy outcomes, such as premature membrane rupture or preterm delivery [11]. Genetic analysis of *T. vaginalis* has revealed the presence of two β-CAs. We have previously reported the production and characterization of the first isoform (TvaCA1) [12–15], and now we describe and characterize the second isoform (TvaCA2) here. These enzymes represent promising target enzymes for antitrichomonal drug development.

## Materials and methods

### Protein production

The gene sequence of the second β-CA of T. vaginalis (*TvaCA2*) was retrieved from Universal Protein Resource Database UniProt (protein entry A2DLG4) [16]. An *Escherichia coli* expression system with a multipromoter vector, pBVboostFG (Online Resource 1), was used for the expression of TvaCA2. Gene synthesis and subcloning were performed by GeneArt (Thermo Fisher Scientific, Waltham, USA). The cells (OneShot® BL21 Star™ (DE3) Chemically Competent Cells, #C601003, Thermo Fisher Scientific, Waltham, USA) were cultured in an orbital shaker (200 rpm, 37 °C) in Luria–Bertani (LB) medium supplemented with 10 mg/mL gentamicin (1:1000). The cells were harvested via centrifugation (4 °C, 15 min, 5000 × *g*) after reaching an optical density of 0.4–0.6, and they were mechanically disrupted with an EmulsiFlex-C3 homogenizer (AVESTIN, Ottawa, Canada) in 50 mM Na_2_HPO_4_, 0.5 M NaCl, and 50 mM imidazole binding buffer (BB; pH 8.0), and centrifuged (13,000 × *g*, 15 min, 4 °C). The supernatant was combined with Protino® nickel-nitrilotriacetic acid (Ni^2+^-NTA) agarose affinity chromatography resin (Macherey–Nagel GmbH Co., Düren, Germany) and BB (1, ≥ 3, *v*/*v*), after which the suspension was incubated for 2 h at room temperature with gentle agitation. After incubation, the resin was packed into a chromatography column with an EMD Millipore™ vacuum filtering flask (#XX1004705, Merck, Darmstad, Germany). The protein was eluted with 50 mM Na_2_HPO_4_, 0.5 M NaCl, and 350 mM imidazole (pH 7.0). The 6xHis-tag was removed by thrombin (#RECOMT, Sigma-Aldrich, St. Louis, USA) according to the Thrombin CleanCleave™ Kit manual (Sigma-Aldrich, St. Louis, USA) followed by Ni^2+^-NTA affinity chromatography. NanoDrop One (Thermo Fisher Scientific, Waltham, USA) was used to determine the protein yield, and the quality of the purified protein was analyzed by SDS-PAGE (sodium dodecyl sulfate–polyacrylamide gel electrophoresis) using a 12% polyacrylamide gel visualized with PageBlue Protein staining solution (Thermo Fisher Scientific, #24,620, Waltham, USA). The obtained polypeptide bands of the SDS-PAGE gel were identified using tandem mass spectrometry (MS/MS; Meilahti Clinical Proteomics Core Facility, University of Helsinki, Finland).

### Multiple sequence alignment

Multiple sequence alignment was performed using the Clustal Omega tool from EMBL-EBI [17] and visualized with Jalview [18]. Sequence identity and similarity were determined by EMBOSS Supermatcher [19].

### Catalytic activity and inhibition assays

CA-catalyzed CO_2_ hydration activity was investigated with an Applied Photophysics stopped-flow instrument at 20 °C [20]. Twenty millimolar Hepes was used as buffer (pH 7.5) with 20 mM Na_2_SO_4_ (for maintaining a constant ionic strength). Phenol red of 0.2 mM was used as a pH indicator, working at a maximum absorbance of 557 nm. The initial rates of the CA-catalyzed CO_2_ hydration reaction were followed for 10–100 s. CO_2_ concentrations of 1.7–17 mM were used to determine the kinetic parameters and inhibition constants. For each inhibitor, the initial velocity was determined by using ≥ 6 traces of the initial 5–10% of the reaction. The uncatalyzed rates were determined similarly and subtracted from the total observed rates. DSMO was used at 5% to make the stock solution, from which 0.1 mM inhibitor solutions were prepared in dH_2_O. Subsequently, dilutions up to 0.01 nM were prepared with dH_2_O. Inhibitor and enzyme solutions were preincubated together (15 min, room temperature) prior to the assay to allow the formation of the enzyme–inhibitor complex. The inhibition constants were obtained by nonlinear least-squares methods using PRISM 3 and represent the means from ≥ 3 different measurements.

### Size exclusion chromatography with light scattering analysis

Static light scattering (SLS) combined with size-exclusion chromatography (SEC) was used to determine the molecular weight (*M*_*w*_) of TvaCA2 without a His-tag. The instrumentation consisted of a Malvern Zetasizer (microV) (Malvern Instruments Ltd., Worcestershire, UK) and a liquid chromatography instrument (CBM-20A, Shimadzu Corporation, Kyoto, Japan) equipped with an autosampler (SIL-20A) and UV–VIS (SPD-20A) and fluorescence detectors (RF-20Axs). The protein concentration was determined with the UV absorption intensity at 280 nm. Lab Solution Version 5.51 (Shimadzu Corporation) and OmniSec 4.7 (Malvern Instruments Ltd., Worcestershire, UK) software were used to process the acquired data. The TvaCA2 sample was injected into a Superdex 200 5/150 column (GE Healthcare, Uppsala, Sweden) equilibrated with 50 mM Tris–HCl (pH 7.5) buffer. Measurements were performed within a thermostable chamber at 12 °C, with a flow rate of 0.1 mL/min. The *M*_*w*_ of TvaCA2 was determined in two independent ways: first, based on elution time by using a standard curve calculated according to the elution profiles of standard proteins (SEC analysis: cytochrome C (CC) 12 kDa, alcohol dehydrogenase 150 kDa (ADH), β-amylase 200 kDa, bovine serum albumin (BSA) 66 kDa, standard CA 29 kDa (Sigma-Aldrich, Inc., St. Louis, MO, USA)) and second, by calibrating the light-scattering detector based on the monomeric peak of BSA and using the SLS intensity to determine the protein size.

### Homology modeling and molecular dynamics

Homology modeling of the TvaCA2 structure was prepared with SWISS-MODEL [21] using the TvaCA1 crystal structure (PDB 6Y04) as a template. Both the homology model of TvaCA1 and the crystal structure of TvaCA1 were equilibrated using molecular dynamics (MD). MD simulations were performed using Gromacs 2021 [22] at the Mahti supercomputer, CSC, Finland. The Amber99SB-ILDN force field with parametrized zinc coordination was used [23–25]. The protein was placed into a dodecahedron box with an explicit SPC/E water model [26]. The total system charge was neutralized using either Na^+^ or Cl^−^ ions. The system was energy minimized using the steepest descent algorithm and equilibrated using harmonic position restraints on all heavy atoms of the protein. An integration time step of 2 fs was used in all the simulations. Bonds with hydrogen atoms were constrained using the LINCS algorithm [27]. A cutoff of 1 nm was applied for the real space and Lennard–Jones interactions. Long-range electrostatics were calculated with the PME method [28]. The temperature and pressure of the system were maintained at 300 K using the V-rescale algorithm [29] with a time constant of 0.1 ps and pressure of 1 bar using the Berendsen algorithm [30] with a time constant of 5 ps. Equilibrium MD simulations were run for 100 ns. The final structure snapshots, captured at 100 ns of the simulations, were used for visualization with PyMOL. The electrostatic surface was calculated using APBS [31].

## Results

### Protein production

**Fig. 1:**
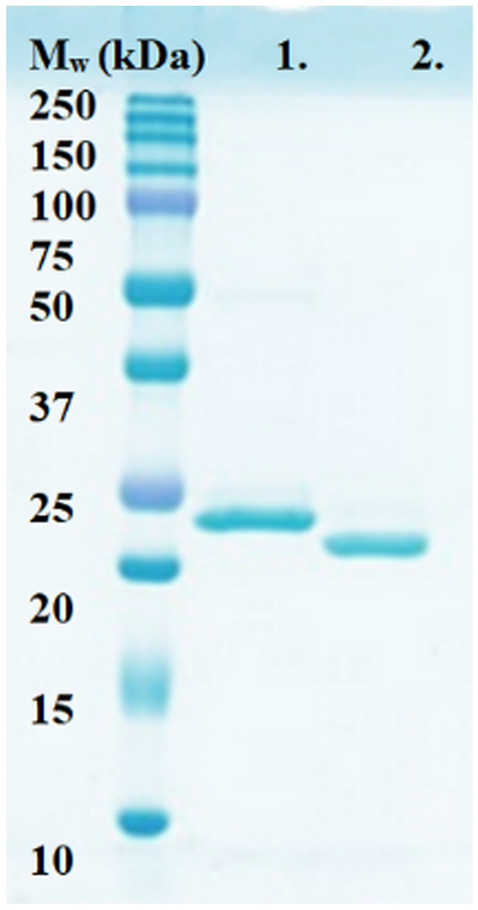
SDS-PAGE image of the cloned and purified TvaCA2 with a 6xHis-tag (1) and without the tag (2). The polypeptide bands visible on the gel were identified as TvaCA2 protein by MS/MS. The standard molecular weight marker (*M*_*w*_) is shown on the far left of the image. The image was stained with PageBlue protein staining solution

TvaCA2 was recombinantly expressed in *E. coli* and purified with affinity chromatography. The yield of the protein was ~ 1 mg of purified protein/L of cell culture. The 6xHis-tag was removed from the purified protein with thrombin, followed by Ni^2+^-NTA purification, and SDS-PAGE. Figure 1 shows that the protein was highly pure and was present as a single polypeptide band under reducing conditions. The *M*_*w*_ of the TvaCA2 band visible in lane 2 was determined to be 20.3 kDa by MS/MS.

### Multiple sequence alignment

**Fig. 2:**
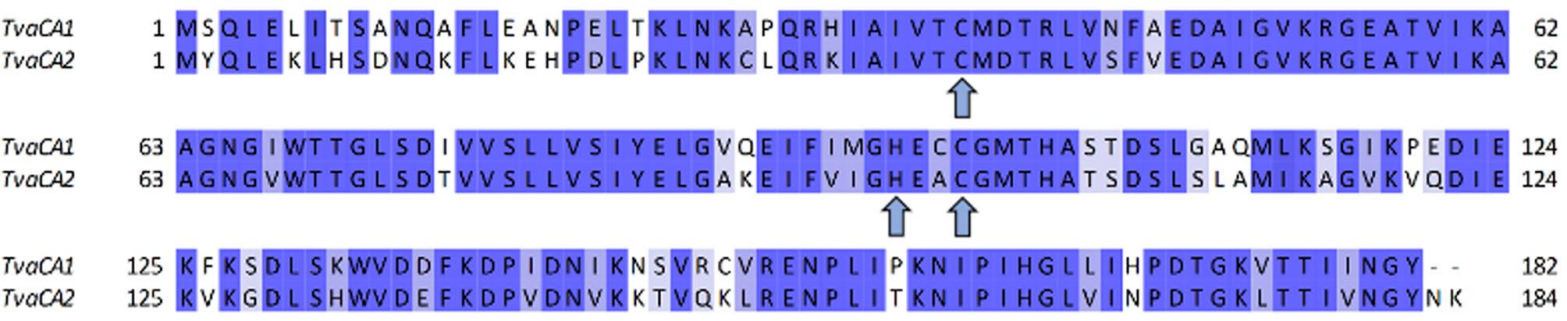
Multiple sequence alignment of TvaCA1 and TvaCA2, determined and visualized with the Jalview bioinformatic analysis web service [18]. The catalytically important zinc ion is coordinated by two cysteines (**C**) and one histidine (**H**) motif, marked with arrows. The aas are shaded with blue; the deepest shade of blue indicates conserved residues, and white indicates differing motifs

Figure 2 shows the multiple sequence alignment of the two proteins. The sequence identity of TvaCA1 and TvaCA2 was 73.1%, and the similarity was 86.8%. Of the amino acids (aas), 144 are fully conserved, 26 are conserved between groups with very similar properties, and 8 are conserved between groups with weakly similar properties. Compared to TvaCA1, TvaCA2 has two additional C-terminal aas (185 aas in TvaCA2 versus 183 aas in TvaCA1). The catalytic zinc ion of these two enzymes is coordinated by two cysteines (Cys37, Cys99) and one histidine (His96).

### Catalytic reaction kinetics

The catalytic activity of TvaCA2 is between that of the highly active human hCA II and the moderately active human hCA I. Therefore, the enzymatic activity of TvaCA2 is considered moderate, with a *k*_cat_ of 3.8 × 10^5^. Similar activities have been reported with the β-CAs of *Candida glabrata* and *Cryptococcus neoformans* [32, 33]. The kinetics of TvaCA1 and TvaCA2 are quite similar, which is apparent due to their high amino acid sequence identity and similarity. When comparing to the kinetics of human CAs, the *k*_cat_ of TvaCA2 showed rather similar values to those of the hCAs, except hCA II, hCA III, hCA IV and hCA IX. Notably, TvaCA2 possessed the same *k*_cat_ as the CA domain of hCA IX expressed in *Escherichia coli* [34].

### Inhibition with sulfonamides

**Fig. 3:**
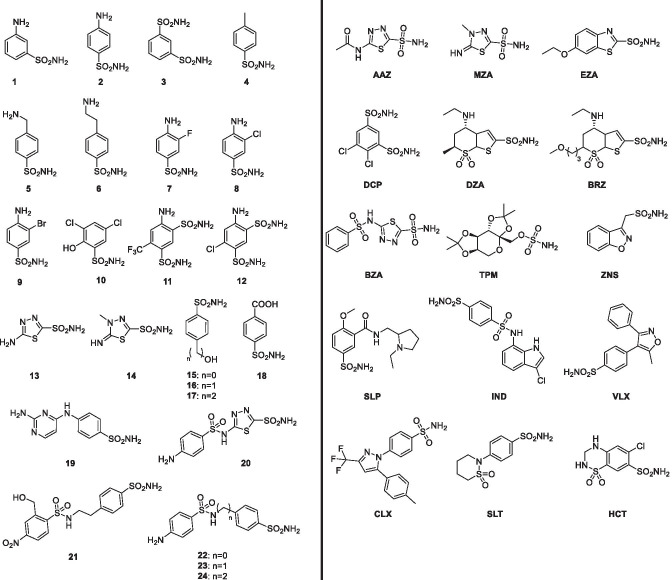
Chemical formulas of the clinically used agents AAZ-HCT and the heterocyclic primary sulfonamides 1–24 studied for the inhibition of TvaCA2

Inhibition of TvaCA2 was investigated with a set of simple/heterocyclic primary sulfonamides 1–24 and the clinically approved drugs AAZ-HCT (chemical formulas shown in Fig. 3).

The following observations can be drawn from the inhibition studies based on the information shown in Table 2:

(i) The most efficient inhibition was established with AAZ, ethoxzolamide (EZA), 4-amino-6-chloro-1,3-benzenedisulfonamide (compound 12), and methazolamide (MZA) with inhibition constants of 222.9 nM, 362.1 nM, 382.2 nM, and 389.8 nM, respectively. From these, AAZ and EZA exhibited similarly strong inhibitions toward both TvaCA1 and TvaCA2.

(ii) Notably, the compounds with a 1,3-benzenedisulfonamide structure (compounds 3, 11, 12, and MZA) were all submicromolar inhibitors of TvaCA2, whereas they were not efficient against TvaCA1.

(iii) The least efficient inhibitors were topiramate (TPM), indisulam (IND), zonisamide (ZNS), sulpride (SLP), celecoxib (CLX), valdecoxib (VLX), sulthiame (SLT), and saccharin (SAC) as well as 3-bromo-4-amino-benzenesulfonamide (compound 9), 1,3-dibenzenesulfonamide (compound 10), 4-(2-amino-pyrimidin-4-yl)-benzenesulfonamide (compound 19), and 4-(2-hydroxymethyl-4-nitrophenyl-sulfonamido)ethylbenzene-sulfonamide (compound 24) with inhibition constants over 10 μM in the assay system, likely reflecting their weak affinity toward TvaCA2.

(iv) Out of the 39 tested sulfonamides 12 compounds resulted in ineffective TvaCA1 inhibition (*K*_*I*_*s* > 10 μM), whereas 25 compounds did not inhibit TvaCA1. In both cases, simple/heterocyclic sulfonamides including 3-bromo-4-amino-benzenesulfonamide (compound 9) and 4-(2-hydroxymethyl-4-nitrophenyl-sulfonamido)ethylbenzene-sulfonamide (compound 24), and the clinical drugs TPM, ZNS, SLP, IND, VLX, CLX, SLT, and SAC exhibited very weak affinities.

(v) Some of the tested compounds resulted in notably different TvaCA2 and TvaCA1 *K*_I_ values. These were 1,3-disulfamoyl benzene (compound 3), 4-amino-6-trifluoromethyl-benzene-1,3-disulfonamide (compound 11), 4-amino-6-chloro-benzene-1,3-disulfonamide (compound 12), MZA, DZA, BRZ, BZA, and HCT, which showed inhibition profiles ranging from strong to moderate/weak TvaCA2 inhibition, whereas with TvaCA1, they resulted in moderate/weak or ineffective inhibition.

As seen in Table 1, the *K*_I_ values of AAZ, EZA, and MZA for TvaCA2 were somewhat similar, in the range of 222.9–389.8 nM. The results of these three classical CA inhibitors were fairly similar between TvaCA1 and TvaCA2, except for MZA which showed approximately 10 times higher inhibition constant (3827.9 nM versus 389.8 nM) for TvaCA1 than for TvaCA2. The other clinically used drugs weakly inhibited both the TvaCA1 and TvaCA2 enzymes with over micromolar inhibition constants. Among compounds 1–24, very few similarities were found in the inhibition profiles of TvaCA1 and TvaCA2. The most similar inhibition constants at micromolar levels were observed for compounds 1 (3-amino-benzenesulfonamide), 13 (5-amino-1,3,4-thiadizole-2-sulfonamide), 15 (4-amino-/4-hydroxymethyl-benzenesulfonamide), and 21 (4-(2-hydroxymethyl-4-nitrophenyl-sulfonamidoethyl)-benzenesulfonamide) with moderate–weak inhibition constants ranging from 1148 to 4155 nM. Compounds 1, 13, and 15 have structures that incorporate compact, simple scaffolds with only two substituents, and compound 21 is an elongated molecule typical of sulfonamylated sulfonamides. The inhibition profiles of TvaCA2 and hCAs are remarkably different, indicating major differences in the active site cavity of these enzymes. This is, in fact, desirable when designing novel drugs against β-CAs that do not have any major off-target effects toward α-CAs. Of the human CAs, only hCA I exhibited a similar inhibition constant toward AAZ as TvaCA2 (250 nM versus 222.9 nM, respectively).

**Table 1:** Kinetic data and inhibition constant with acetazolamide (AAZ) of TvaCA2. Similar data of the human α-CA isozymes I and II, TvaCA1, and β-CAs of other pathogens are also presented for comparison [12, 33, 35, 36]

Enzyme	Class	*k*_cat_ (s^−1^)	*k*_cat_/*K*_*M*_ (M^−1^ s^−1^)	*K*_I_ (AAZ, nM)
hCA I [36]	α	2.0 × 10^5^	5.0 × 10^7^	250
hCA II [36]	α	1.4 × 10^6^	1.5 × 10^8^	12
TvaCA1 [12]	β	4.9 × 10^5^	8.0 × 10^7^	391
TvaCA2	β	3.8 × 10^5^	4.4 × 10^7^	222.9
CglCA [32]	β	3.8 × 10^5^	9.7 × 10^7^	11
CanCA [33]	β	3.9 × 10^5^	4.3 × 10^7^	10.5

CglCA β-carbonic anhydrase of Candida glabrata, CanCA β-carbonic anhydrase of Cryptococcus neoformans

**Table 2:** Inhibition data of TvaCA2, TvaCA1, and hCA II with heterocyclic primary sulfonamides 1–24 and the clinically used drugs AAZ-HCT [12, 37]

*K*_I_ (nM)*			
Inhibitor/enzyme class	TvaCA2 β	TvaCA1 [12] β	hCA II [37] α
01	3925.2	3245.8	300
02	1295.0	4742.1	240
03	446.1	3559.3	8
04	1295.0	3599.1	320
05	4187.5	> 10,000	170
06	4228.2	> 10,000	160
07	1295.0	4282.3	60
08	3954.0	> 10,000	110
09	> 10,000	> 10,000	40
10	> 10,000	4536.2	54
11	440.3	> 10,000	63
12	382.2	> 10,000	75
13	1147.9	1889.2	60
14	1615.0	3987.1	19
15	2333.3	2026.7	80
16	1895.0	> 10,000	94
17	1378.7	> 10,000	125
18	1632.2	> 10,000	46
19	> 10,000	4528.7	33
20	1043.1	> 10,000	2
21	4155.1	3450.0	11
22	3904.3	> 10,000	46
23	2258.6	> 10,000	33
24	> 10,000	> 10,000	30
AAZ	222.9	391.3	12
MZA	389.8	3827.9	14
EZA	362.1	283.6	8
DZA	1695.3	> 10,000	9
BRZ	3230.0	> 10,000	3
BZA	4206.8	> 10,000	9
TPM	> 10,000	> 10,000	10
ZNS	> 10,000	> 10,000	35
SLP	> 10,000	> 10,000	40
IND	> 10,000	> 10,000	15
VLX	> 10,000	> 10,000	43
CLX	> 10,000	> 10,000	21
SLT	> 10,000	> 10,000	9
SAC	> 10,000	> 10,000	5959
HCT	3061.8	> 10,000	290

*Mean from three different assays, obtained by a stopped flow technique. Errors were in the range of ± 5–10% of the reported values

### Determination of oligomeric state using chromatography and light scattering

SEC-SLS was used to investigate the quaternary structure of the purified TvaCA2. A total of three different measurements were performed with TvaCA2 samples. All measurements were done in a thermostable chamber at 12 °C. Measured retention volumes and molecular weights of TvaCA2 and standard protein samples (CC, ADH, β-amylase, BSA, and CA) are presented in Table 3.

**Table 3:** Elution profiles of measured for TvaCA2 and standard protein samples

Sample	Retention volume (ml)	Molecular weight (kDa)
CC	3.3	10.3
ADH	1.7	130.9
β-Amylase	1.6	272.1
BSA	1.8	66.6
Standard CA	2.2	29.3
TvaCA2	2.2	44.3

**Fig. 4:**
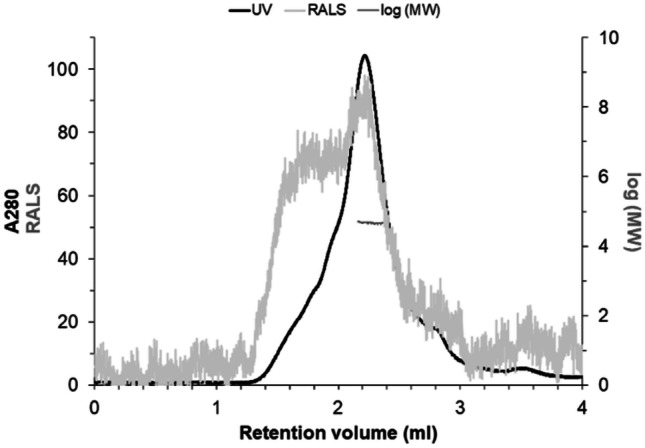
Light scattering data for the assessment of the oligomeric state and size of TvaCA2. The *Y*-axis on the left shows the UV absorption intensity at 280 nm and the arbitrary right-angle light scattering intensity (RALS). The *X*-axis on the right shows the *M*_*w*_ calculated using static LS intensity. The *X-*axis shows the retention volume of the protein

Figure 4 is a representative image of the LS data. UV absorption at 280 nm (black curve) indicates when the main peak was eluted. The protein size of TvaCA2 was determined by two methods. First, the standard curve was used to obtain an *M*_*w*_ estimation of 46 kDa. Second, the SLS intensity was used to obtain an *M*_*w*_ estimation of 44.3 kDa (the horizontal dark gray line across the main peak). For comparison, MS/MS revealed a *M*_*w*_ of 20.45 kDa for the monomeric TvaCA2, making the dimeric form 40.9 kDa. Additionally, based on the primary sequence, Expasy [38] gave an estimate of 20.3 kDa for the monomer of TvaCA2 and therefore a calculated estimation of 40.6 kDa for the dimer. Even though the *M*_*w*_ values determined with analytical gel filtration and SEC-SLS differed to some extent from those obtained from MS/MS and Expasy, it can be concluded that TvaCA2 is dimeric in solution, similar to the first isoform [12].

### Molecular modeling

Homology modeling was used to predict the structure of TvaCA2, using the TvaCA1 crystal structure (PDB 6Y04) as a template [12]. Subsequently, MD simulations were used to equilibrate both the TvaCA1 crystal structure and the TvaCA2 model, using 100-ns unrestrained dynamics. Both the root mean square deviations and root mean square fluctuations (Online Resource 2a, b) of backbone atoms showed similar flexibility of the enzymes, indicating the model was high quality.

**Fig. 5:**
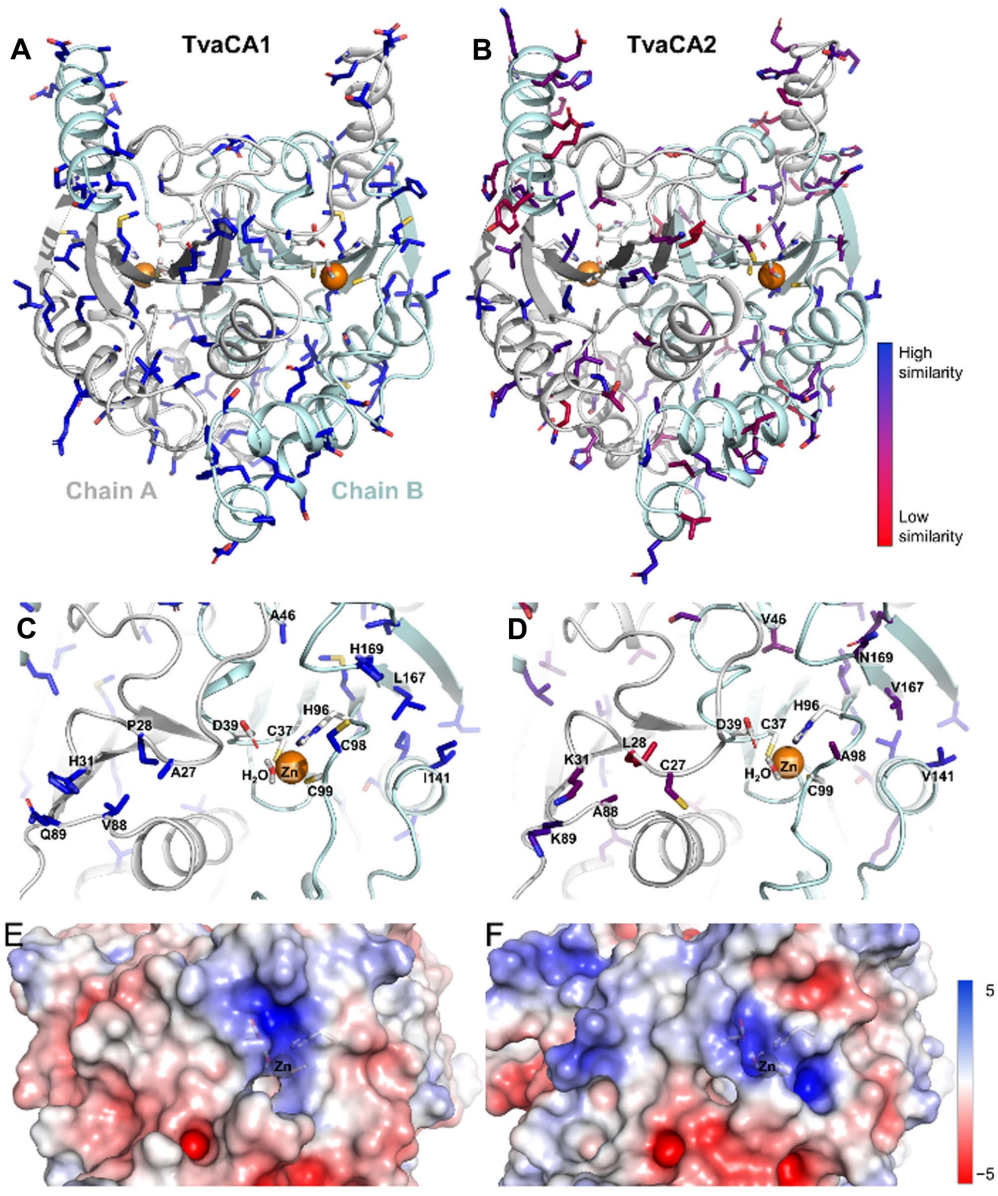
Structural differences between TvaCA1 and TvaCA2. (**a**) Cartoon representation of MD-equilibrated crystal structure (PDB 6Y04) TvaCA1 and (**c**) its active site, where the residues that are different from TvaCA2 are shown as blue sticks. (**b**) Cartoon representation of the MD-equilibrated homology model of TvaCA2 and (**d**) its active site, where the residues that are different from TvaCA1 are shown as sticks and colored from blue to red, depending on similarity to the corresponding residue in TvaCA1 according to the BLOSUM90 substitution matrix. Zinc ion is shown as an orange sphere. The electrostatic surfaces of (**e**) TvaCA1 and (**f**) TvaCA2 in the active site region are shown

TvaCA2 has a similar structure to TvaCA1 (Fig. 5(a, b)), forming a homodimer with a β-sheet core. Both enzymes have two active sites, where a zinc ion is coordinated by Cys37, His96, and Cys99. MD simulations suggest that H_2_O is present in each active site, interacting with Zn^2+^ and Asp39 (Fig. 5(c, d)). The electrostatic surface of the active site for TvaCA1 and TvaCA2 is shown in Fig. 5 (e, f), and the surface of the whole enzyme is shown in Online Resource 2c, d.

## Discussion and conclusions

*Trichomonas vaginalis*, a protozoan parasite and a causative agent of one of the most common STIs, trichomoniasis, has two β-carbonic anhydrases. The first has been characterized earlier by us [12–15], and the second isoform has been presented here. These isozymes are of particular interest because they represent potential novel targets for antitrichomonal drugs. Antibiotic-resistant pathogens are an emerging global concern, instigating the need for novel medications. The first antibiotic-resistant *T. vaginalis* emerged in 1981 [39]. Since then, increasing number of refractory cases has been reported. Even though trichomoniasis causes mild–severe symptoms^1^, it is a disease that requires serious consideration. Patients with mild/no symptoms are accountable for spreading the infection, and today, trichomoniasis accounts for nearly half of all STIs worldwide. Trichomoniasis infections lead to increased susceptibility to HIV acquisition and/or transmission [9], adverse pregnancy outcomes [11], and progression of prostate cancer [10]. Currently, the only cure is antibiotics, often involving multiple rounds of medication that lead to a lack of drug effectiveness and increased antibiotic resistance [7]. CAs have been presented as promising novel therapeutic targets, since humans have only *α-CA* genes and many clinically significant pathogens have *β-* and/or *γ-CA* genes in their genome.

The first step of CA-related drug design involves the identification, production, and characterization of pathogen-specific CAs. With these data, it is possible to further develop and test β- and/or γ-CA specific inhibitors that block the catalytic function of these enzymes. Since CAs play vital roles in many biochemical processes in all living organisms, inhibiting them could ultimately lead to elimination of target pathogens. This has already been studied with promising results in several pathogenic microorganisms (MOs). For instance, experiments involving *Leishmania donovani chagasi. Plasmodium falciparum*, *Brucella suis*, *Helicobacter pylori*, *Candida albicans*, and *Cryptococcus neoformans* showed that inhibiting the CAs of these MOs in vivo resulted in growth impairment, death of the MOs, and/or decrease of their virulence. [4, 40–44] This has confirmed the druggability of β- and/or γ-CAs from pathogens and encourages further investigations to find more potent inhibitors. The differences between α- and β-/γ-CAs in protein structures, kinetics, inhibition, and activation profiles enable drug development aiming to minimize off-target effects, which as we know, is one of the challenges related to current antibiotics.

In this study, TvaCA2 was successfully expressed in *E. coli* and purified with affinity chromatography. On SDS-PAGE gel, TvaCA2 was present as a single polypeptide with an *M*_*w*_ of 20.3 kDa (determined by MS/MS). LS analysis revealed that the native form of TvaCA2 is dimeric in solution, which was predicted since the first characterized β-CA of *T. vaginalis* (TvaCA1) was previously determined to be a dimer by X-ray crystallography [12]. There was 73.1% sequence identity and 86.8% similarity between TvaCA1 and TvaCA2. Multiple sequence alignment revealed that in addition to the active site, these proteins share high similarity throughout the sequence.

Inhibition of TvaCA2 was studied with a set of simple/heterocyclic primary sulfonamides 1–24 and several clinically approved drugs, which showed CA inhibition properties. The most successful inhibition was established with AAZ, EZA, 4-amino-6-chloro-1,3-benzenedisulfonamide (compound 12), and MZA with inhibition constants of 222.9 nM, 362.1 nM, 382.2 nM, and 389.8 nM, respectively. The 1,3-benzenedisulfonamide structure present in 1,3-disulfamoyl benzene (compound 3), 4-amino-6-trifluoromethyl-benzene-1,3-disulfonamide (compound 11), and 4-amino-6-chloro-benzene-1,3-disulfonamide (compound 12) appeared to be a promising scaffold for drug design against TvaCA2, whereas these compounds were inefficient against TvaCA1. These inhibitors incorporate compact scaffolds, which should be able to enter the long and narrow active site of both *T. vaginalis* enzymes. In particular, among compounds 1–24, very few similarities were found between the inhibition profiles of TvaCA1 and TvaCA2. The differing results may be due to different charge distributions and differences in the protein surface architecture near the active sites of TvaCA1 and TvaCA2. Overall, out of the 39 tested sulfonamides, 12 compounds remained ineffective against TvaCA2, and 25 remained ineffective against TvaCA1. Most of these inhibitors possess a bulky scaffold, with various substituents on which the sulfonamide or sulfamate moieties are attached. These features most likely interfere with their efficient binding within the long, channel-like active site of TvaCA1 or TvaCA2, explaining their poor inhibition capacity.

To learn about the mechanisms behind the different inhibition profiles, we built a 3D model of TvCA2. In silico homology modeling suggested that TvaCA2 and TvaCA1 have very similar protein structures. Both enzymes have Cys37, His96, and Cys99 within the active cavity, where H_2_O interacts with Zn^2+^ and Asp39. Inspection of the vicinity of the active site revealed features of interest, which may explain the differences in inhibition profiles. For example, TvCA2 showed different charge distributions and there were differences in the protein surface architecture near the active site, potentially reflecting inhibitor specificity and affinity. More detailed studies are needed to develop high-affinity inhibitors for TvaCAs, and the current study provides a good starting point for such work.

## Supplementary Information

Below is the link to the electronic supplementary material.

Supplementary file1 (DOCX 1926 KB)

## Data Availability

The authors confirm that the data supporting the findings of this study are available within the article and its supplementary materials.
