# Supplementary material for: Biochemical and structural characterization of beta-carbonic anhydrase from the parasite Trichomonas vaginalis
Source: J Mol Med (Berl). 2021 Oct 15;100(1):115–24. doi: 10.1007/s00109-021-02148-1 (PMC8724216; doi:10.1007/s00109-021-02148-1)
Supplement: Supplementary file 1 — Supplementary file1 (DOCX 1926 KB) [file 109_2021_2148_MOESM1_ESM.docx]

Biochemical and structural characterization of beta-carbonic anhydrase from the parasite *Trichomonas vaginalis*

Linda J. Urbański^a^*, Andrea Angeli^b^, Vasyl V. Mykuliak^a^, Latifeh Azizi^a^, Marianne Kuuslahti^a^, Vesa P. Hytönen^a,c^‡, Claudiu T. Supuran^b^‡, and Seppo Parkkila^a,c^‡

^a^Faculty of Medicine and Health Technology, Tampere University, Arvo Ylpön katu 34, FI-33520 Tampere Finland

^b^Neurofarba Department, Sezione di Chimica Farmaceutica e Nutraceutica, Università degli Studi di Firenze, Via U. Schiff 6, I-50019 Sesto Fiorentino (Firenze), Italy

^c^Fimlab Ltd, Tampere University Hospital, Arvo Ylpön katu 4, FI-33520 Tampere, Finland

* Corresponding author: linda.urbanski@tuni.fi

‡ Equal contribution as senior authors.


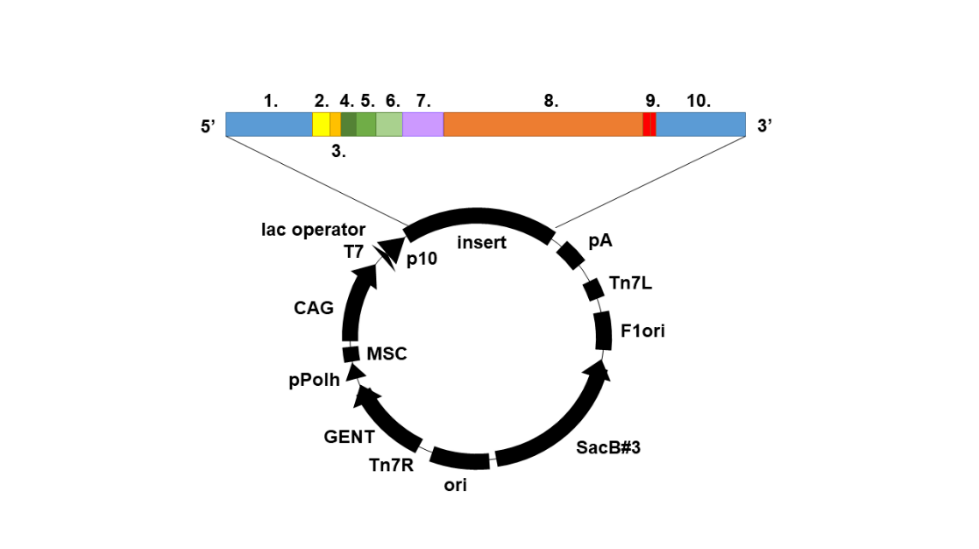


**Online resource 1** Illustration of the pBVboostFG expression vector. The designed parts of the insert: 1. attL1, 2. Shine-Dalgarno, 3. Kozak, 4. Met-Ser-Tyr-Tyr, 5. 6 x His, 6. Asp-Tyr-Asp-Ile-Pro-Thr-Thr, 7. Thrombin cleavage site (Lys-Val-Pro-Arg-Gly-Ser [1]), 8. CA gene of interest, 9. 2 x stop codon, 10. attL2


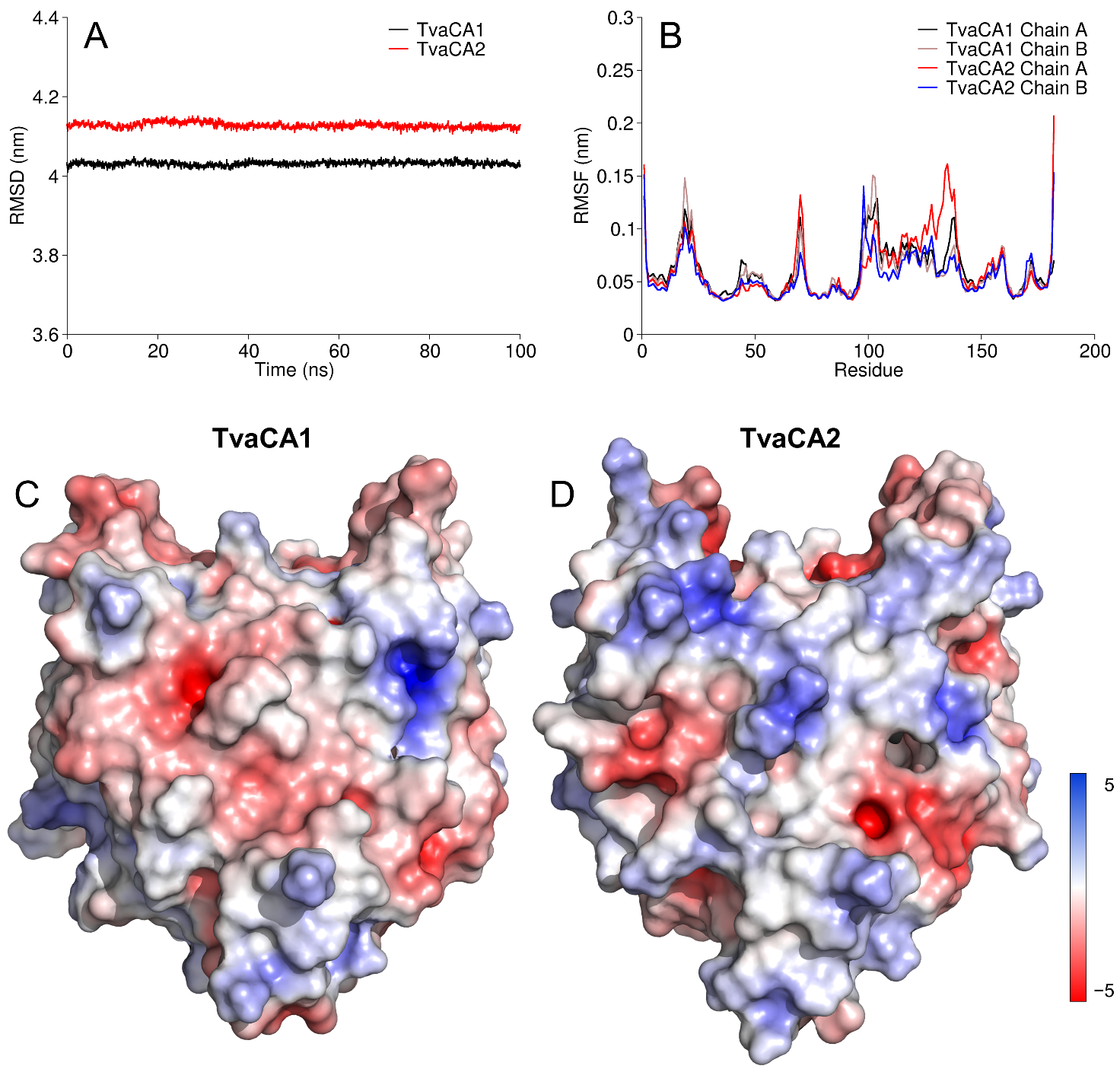


**Online resource 2** Electrostatic surface and backbone flexibility in MD. (a) Root Mean Square Deviations and (b) Root Mean Square Fluctuations of backbone atoms in the MD trajectories. The electrostatic surface of (c) TvaCA1 and (d) TvaCA2. Root Mean Square Fluctuations were calculated for 20 – 100 ns of the MD trajectories

REFERENCES

1. Hilvo, M.; Baranauskiene, L.; Salzano, A. M.; Scaloni, A.; Matulis, D.; Innocenti, A.; Scozzafava, A.; Monti, S. M.; Di Fiore, A.; De Simone, G.; Lindfors, M.; Janis, J.; Valjakka, J.; Pastorekova, S.; Pastorek, J.; Kulomaa, M. S.; Nordlund, H. R.; Supuran, C. T.; Parkkila, S., Biochemical characterization of CA IX, one of the most active carbonic anhydrase isozymes. *J Biol Chem* **2008,** 283, (41), 27799-809.
